# Retraction Note: Decreased microRNA-452 expression and its prognostic significance in human osteosarcoma

**DOI:** 10.1186/s12957-019-1648-y

**Published:** 2019-06-14

**Authors:** Ren-Zeng Li, Li-Min Wang

**Affiliations:** 1 Department of Orthopedics, The First Affiliated Hospital of Zhengzhou University, Zhengzhou, 450000 Henan Province People’s Republic of China; 2 Department of Orthopedics, People No.3 Hospital of Anyang, Anyang, 455000 Henan Province People’s Republic of China

**Retraction note: World J Surg Oncol**

**https://doi.org/10.1186/s12957-016-0900-y**

The Editor-in-Chief is retracting this article [1] due to overlap with previously published articles [2, 3]. Fig. 3D “miR-452 mimics” is very similar to Fig. 3D “si-BANCR” in article [2]. Fig. 3D “miRNC” is very similar to Fig. 3E “si-NC” in article [2] and to Fig. 3E “miR-224 mimics” in article [3].

None of the authors responded to any correspondence from the Editor-in-Chief or publisher regarding this retraction.
